# Long pentraxin 3 (PTX3) levels predict death, intubation and thrombotic events among hospitalized patients with COVID-19

**DOI:** 10.3389/fimmu.2022.933960

**Published:** 2022-10-28

**Authors:** Giuseppe Lapadula, Roberto Leone, Davide Paolo Bernasconi, Andrea Biondi, Emanuela Rossi, Mariella D’Angiò, Barbara Bottazzi, Laura Rachele Bettini, Ilaria Beretta, Cecilia Garlanda, Maria Grazia Valsecchi, Alberto Mantovani, Paolo Bonfanti

**Affiliations:** ^1^ School of Medicine and Surgery, University of Milano-Bicocca, Milan, Italy; ^2^ Department of Infectious Diseases, San Gerardo Hospital, Monza, Italy; ^3^ IRCCS Humanitas Research Hospital, Milan, Italy; ^4^ Bicocca Bioinformatics Biostatistics and Bioimaging Center - B4, University of Milano–Bicocca, Milan, Italy; ^5^ Department of Pediatrics, European Reference Network (ERN) PaedCan, EuroBloodNet, MetabERN Fondazione Monza e Brianza per il Bambino e la sua Mamma (MBBM)/Ospedale San Gerardo, Monza, Italy; ^6^ Department of Biomedical Sciences, Humanitas University, Pieve Emanuele, Italy; ^7^ William Harvey Research Institute, Queen Mary University, London, United Kingdom

**Keywords:** COVID - 19, pentraxin 3 (PTX3), mortality, SARS – CoV – 2, inflammation

## Abstract

**Background:**

PTX3 is an important mediator of inflammation and innate immunity. We aimed at assessing its prognostic value in a large cohort of patients hospitalized with COVID-19.

**Methods:**

Levels of PTX3 were measured in 152 patients hospitalized with COVID-19 at San Gerardo Hospital (Monza, Italy) since March 2020. Cox regression was used to identify predictors of time from admission to in-hospital death or mechanical ventilation. Crude incidences of death were compared between patients with PTX3 levels higher or lower than the best cut-off estimated with the Maximally Selected Rank Statistics Method.

**Results:**

Upon admission, 22% of the patients required no oxygen, 46% low-flow oxygen, 30% high-flow nasal cannula or CPAP-helmet and 3% MV. Median level of PTX3 was 21.7 (IQR: 13.5-58.23) ng/ml. In-hospital mortality was 25% (38 deaths); 13 patients (8.6%) underwent MV. PTX3 was associated with risk of death (per 10 ng/ml, HR 1.08; 95%CI 1.04-1.11; P<0.001) and death/MV (HR 1.04; 95%CI 1.01-1.07; P=0.011), independently of other predictors of in-hospital mortality, including age, Charlson Comorbidity Index, D-dimer and C-reactive protein (CRP). Patients with PTX3 levels above the optimal cut-off of 39.32 ng/ml had significantly higher mortality than the others (55% vs 8%, P<0.001). Higher PTX3 plasma levels were found in 14 patients with subsequent thrombotic complications (median [IQR]: 51.4 [24.6-94.4] *versus* 21 [13.4-55.2]; P=0.049).

**Conclusions:**

High PTX3 levels in patients hospitalized with COVID-19 are associated with a worse outcome. The evaluation of this marker could be useful in prognostic stratification and identification of patients who could benefit from immunomodulant therapy.

## Background

Coronavirus Diseases-19 (COVID-19), caused by severe acute respiratory syndrome coronavirus-2 (SARS-CoV-2), spread all over the world starting from China at the end of 2019 (1, 2). The disease is characterized by an heterogeneous set of manifestations that vary from self-resolving asymptomatic infections to fatal acute respiratory distress (ARDS) (3). Even among patients requiring hospitalization, it is often difficult, at presentation, to predict the evolution towards a severe or critical form, which put patient’s life at risk despite supportive care. Laboratory markers capable of identifying people at greatest risk of unfavorable outcome would be of invaluable help in patient management.

The inflammatory response is of utmost importance as a front-line defense against viruses and to trigger the subsequent activation of innate immunity. On the other hand, however, excessive immune-activation and hyper-inflammation can contribute to the pathogenesis, morbidity and mortality associated with viral infections. In agreement, the infection with SARS-CoV-2 is associated with a massive inflammatory activation as well as vasculopathy, endothelial dysfunction and thrombotic complication (4–7). The identification of biomarkers predicting mortality risk of Covid-19 patients thus represents a still unmet medical need to improve the management of these patients.

The long pentraxin 3 (PTX3) is an important mediator of inflammation and innate immunity, and a cognate molecule of C-reactive protein (CRP) (8, 9). PTX3 is expressed by myeloid and stromal cells, in particular endothelial and epithelial cells, in response to primary proinflammatory signals, TLR engagement, microbial recognition and tissue damage (8, 10, 11). The main characteristics of PTX3 are the local production at the site of infection or tissue damage by multiple cell types, and the consequent prompt increase of its circulating levels in inflammatory or infectious conditions, which make this molecule a candidate prognostic biomarker. In various pathological conditions, ranging from cardiovascular diseases to infections and sepsis, PTX3 plasma levels are increased and generally correlated with severity (12–17). Enhanced expression of PTX3 and correlation with disease activity has been also reported in conditions of vascular inflammation, including giant cell arteritis, Takayasu’s arteritis, and ANCA-associated small vessel vasculitis (18–21). In addition, plasma levels of PTX3 have been reported to predict mortality in several systemic inflammatory conditions, including acute myocardial infarction (22, 23) and sepsis (24–27). Overall, these observations led to evaluate the role of PTX3 as prognostic biomarker of SARS-CoV-2 infections.

In a cohort of 96 patients with COVID-19, PTX3 levels measured at hospital admission were significantly increased compared to healthy controls and were predictors of 28-days mortality (28). These data were confirmed by other reports, all performed on small cohorts including 39 to 126 patients (29–33). Recently PTX3 has also emerged as strongly associated with long COVID syndrome (34).

In this scenario we decided to further analyse the prognostic value of PTX3 in a larger cohort of patients hospitalized with COVID-19 with the aim to extend the observations to a randomly selected population from an observational cohort, increasing its representativeness. The results further confirmed the possible use of PTX3 as a risk marker of clinical progression, and for the first time associated PTX3 levels with the thrombotic complications observed in Covid-19 patients.

## Methods

### Study population

The STORM Study is a prospective observational cohort which enrolled consecutive patients hospitalized with COVID-19 in the “San Gerardo” Hospital (Monza, Italy). “San Gerardo” is the reference hospital for COVID-19 of one of the most densely populated area in Italy (Brianza, Lombardy), which was affected by SARS-CoV-2 circulation since early 2020 and borders with the Bergamo area, epicenter of the spread of the epidemic in Italy.

Information on patients’ demographics, comorbidities, laboratory results, treatment history and disease course were recorded. Plasma samples were collected at hospital presentation or within 48 hours of admission and stored at -80°C. The study was approved by the Italian Institutional Review Board for COVID-19 Studies and all patients signed an informed consent before inclusion in the cohort. The patients included in the present study were enrolled during the “first wave” of the pandemic, between March 16th and May 31st, 2020 and were a random selection of those who had a plasma sample available.

### Pentraxin 3 analysis

PTX3 was quantified in plasma samples collected from patients at their admission to “San Gerardo” and stored at -80°C until use. Levels were measured by a sandwich enzyme-linked immunosorbent assay (ELISA) developed in Humanitas Clinical and Research Institute by personnel blind to patients’ characteristics, in according to previously described procedures (35). Briefly, plates were coated with MNB4 monoclonal antibody anti human PTX3 in 15mM carbonate buffer pH 9.6. After blocking of non-specific sites with 5% dry milk, two dilutions in duplicate of plasma samples were added and incubated at 37°C for 2 hours in parallel with a standard curve of recombinant PTX3 (range 0.075-2.4 ng/ml). A rabbit antiserum affinity purified on human PTX3 and biotinylated (pAb) was used to detect bound PTX3. After incubation with horse-radish conjugated streptavidin followed by the TMB (3,3’,5,5’-tetramethylbenzidine) chromogen, absorbance at 450nm was measured with an automatic ELISA reader and PTX3 content in plasma samples was calculated based on the standard curve with the recombinant protein. The assay has a detection limit of 0.1 ng/ml and an inter-assay variability from 8 to 10%. Each sample was tested in duplicate. No cross reaction of MNB4 and pAb with human CRP and serum amyloid P component protein has been observed.

### Statistical analysis

Patients’ characteristics at admission were described using median (IQR) for continuous variables and absolute numbers and proportions for categorical variables. Uni- and multivariable Cox regression models were fitted to identify predictors of two time-to-event endpoints: time from admission i) to in-hospital death (discharge was considered as a competing risk) and ii) to in –hospital death or need for mechanical ventilation (discharge before mechanical ventilation was considered as a competing risk). The following covariates were included in the models: age, gender, respiratory support at admission, Charlson Comorbidity Index, lymphocyte count, levels of PTX3, C-reactive protein (CRP) and D-dimer. Crude incidences of the outcomes of interest were estimated using Aalen-Johansen methods and compared using Grey test between patients with PTX3 levels higher or lower than the best cut-off. This threshold was estimated according to the Maximally Selected Rank Statistics Method (36). The additional discriminatory ability attributable to PTX3, on top of the other predictors, was estimated as the difference of cross-validated c-statistic between the multivariable models with and without PTX3 as covariate. Cross-validation was performed using 150 bootstrap replicates.

## Results

One hundred fifty-two patients were analyzed. They were predominantly male (62%) and Caucasian (94%); their median age was 67 years (interquartile range [IQR]: 55-80). Upon admission, 22% did not require oxygen supplementation, 46% required oxygen delivered through nasal cannula (NC) or face mask, 30% were treated with non-invasive ventilation (NIV) using continuous positive airway pressure (CPAP) helmet and 3% needed mechanical ventilation (MV). **Table 1** shows these and other patient characteristics at the time of hospital admission in the overall population and grouped by clinical outcome (mechanical ventilation/deceased *versus* survivors).

**Table 1:** Characteristics and outcomes of the patients included in the study.

Patient characteristics	Overall(N=152)	Died or mechanically ventilated(N=47)	Discharged alive free from MV(N=105)	p-value
Male gender, N (%)	94 (61.8)	32 (68.1)	62 (59)	0.379
Age, in years (Median [IQR])	67 [55-80]	75 [67-86]	62 [52-76]	<0.001
Ethnicity, N (%) Caucasian Hispanic/South American Other/Unknown	143 (94.1) 5 (3.3) 4 (2.6)	47 (100.0) 0 0	96 (91.4) 5 (4.8) 4 (3.8)	0.369
Charlson Comorbidity Index (Median [IQR])	4 [1-5]	4 [4-6]	3 [1-5]	<0.001
Respiratory support at admission No oxygen Low-flow nasal cannula Oxygen mask CPAP Helmet or high-flow nasal cannula Mechanical Ventilation	33 (21.7) 46 (30.3) 23 (15.2) 46 (30.3) 4 (2.6)	4 (8.5) 5 (10.6) 12 (25.5) 22 (46.8) 4 (8.5)	29 (27.6) 41 (39) 11 (10.5) 24 (22.8) 0	<0.001
Lymphocyte count, x10^3/μl (Median [IQR]) **	1.05 [0.72-1.38]	0.74 [0.57-1.10]	1.18 [0.85-1.49]	<0.001
D-dimer, ng/ml (Median [IQR])*	403 [223-1089]	745 [350-2187]	340 [192-684]	<0.001
C-reactive protein, mg/dl (Median [IQR]) **	8.7 [2.6-14.6]	13.8 [6.8-20.1]	4.3 [1.2-10.9]	<0.001
Pentraxin 3, ng/ml (Median [IQR])	21.7 [13.5, 58.2]	68.3 [39.8-115]	18 [11.1-29.1]	<0.001
Vascular events, N (%) Pulmonary embolism Deep Vein Thrombosis Arterial Thrombosis	6 (3.9) 10 (6.6) 1 (0.7)	4 (8.5) 8 (17.0) 0	2 (1.9) 2 (1.9) 1 (1.0)	0.138 0.002 1
In-hospital outcome, N (%) Dead Discharged alive Transferred out	38 (25) 92 (60.5) 22 (14.5)	38 (80.8) 6 (12.8) 3 (6.4)	0 86 (81.9) 19 (18.1)	–

* Available for 124 patients; ** Available for 148 patients.

CPAP, continuous airway positive pressure; IQR, interquartile range; MV, mechanical ventilation; N, number.

Patients were observed for a median of 12 days (IQR: 6-20). During hospital stay, the highest oxygen requirement was low-flow oxygen, high-flow oxygen (i.e., >10 l/min) or NIV and intubation in 41.4%, 32.2%, and 8.6%, respectively. 17.8% did not require any oxygen supplementation at any time during hospitalization. One hundred and five (69.1%) patients were discharged or transferred out free from mechanical ventilation after a median of 12 days (IQR: 7-19); 36 patients (23.7%) died at a median time of 9 days (IQR: 4-16) since admission.

**Figure 1:**
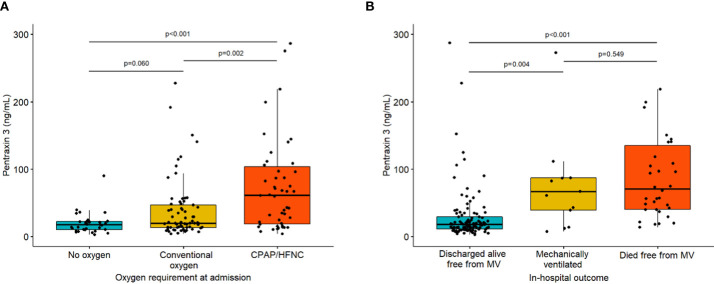
Baseline PTX3 levels according to oxygen requirement at admission (Panel **A**) and in-hospital outcome (panel **B**). P-values are from pairwise Mann-Whitney tests with Holm correction for multiplicity. List of abbreviations: MV, mechanical ventilation.

Levels of PTX3 were measured on samples collected at a median of 1 days (IQR: 1-2) since admission. Median (IQR) level of PTX3 was 21.7 (13.5-58.23) ng/ml. As shown in **Table 1** and **Figure 1**, PTX3 levels at baseline were significantly higher in those who died or needed MV during the hospitalization than among survivors (68.3 *versus* 18 ng/ml, P<0.001). Of note, comparable PTX3 levels were measured among those who had access to mechanical ventilation as compared with those who died free from it (**Figure 1B**).

Levels of PTX3 were moderately correlated with CRP levels (r=0.48, p<0.001) and weakly correlated with D-dimer levels (r=0.25, p=0.005). A complete correlation matrix between laboratory markers is provided as **Supplementary Figure 1**.

Using univariable Cox regression (**Table 2**), PTX3 was associated with the hazard of death (per 10 ng/ml increase, hazard ratio (HR) 1.07; 95%CI 1.04-1.09; P<0.001). Other predictors of in-hospital mortality were older age (HR per year 1.05: 95%CI 1.02-1.07, P<0.001), Charlson Comorbidity Index (HR per unit 1.26; 95%CI 1.13-1.41, P<0.001), D-dimer (HR per 1000 IU/ml 1.17; 95%CI 1.04-1.36, P=0.011) and CRP (per 10 mg/dl increase, HR 1.73; 95%CI 1.24-2.41; P=0.001).

**Table 2:** Uni- and multivariable cause-specific Cox regression models for the two endpoints of interest.

Variable	Outcome: Death		Outcome: Death or Mechanical Ventilation	
	Univariate analysisHR (95%CI)*P*	Multivariable analysisHR (95%CI)*P*	Univariate analysisHR (95%CI)*P*	Multivariable analysisHR (95%CI)*P*
Age (per year)	1.05 (1.02-1.07) *<0.001*	1.06 (1.01-1.11) *0.018*	1.02 (1.00;1.04) *0.027*	1.02 (0.99;1.06) *0.215*
Male gender	1.24 (0.63-2.43) *0.539*	1.25 (0.52-2.98) *0.619*	1.63 (0.88;3.03) *0.123*	1.46 (0.7;3.03) *0.309*
Charlson Index (per 1 unit)	1.26 (1.13-1.41) *<0.001*	1.20 (1.01-1.42) *0.035*	1.17 (1.06;1.29) *0.002*	1.12 (0.96;1.31) *0.160*
Baseline oxygen support No oxygen Low-flow High-flow/CPAP/MV	1 1.83 (0.61-5.45) *0.277* 2.05 (0.69-6.14) *0.198*	1 1.00 (0.25-4.04) *0.995* .16 (0.26-5.13) *0.847*	1 1.88 (0.63;5.60) *0.255* 4.75 (1.66;13.62) *0.004*	1 1.08 (0.29;4.08) *0.911* 2.14 (0.56;8.14) *0.265*
Lymphocytes (per 10^3/µl)	0.88 (0.54-1.44) *0.609*	1.15 (0.95-1.38) *0.153*	0.58 (0.32;1.07) *0.084*	1.03 (0.81;1.29) *0.838*
D-dimer (per 1000 IU/ml)	1.17 (1.04-1.36) *0.011*	1.14 (0.94-1.39) *0.188*	1.24 (1.11;1.38) *<0.001*	1.06 (0.91;1.23) *0.469*
CRP (per 10 mg/dl)	1.73 (1.24-2.41) *0.001*	1.68 (0.99-2.87) *0.057*	2.65 (1.90;3.70) *<0.001*	2.04 (1.21;3.46) *0.008*
PTX3 (per 10 ng/ml)	1.07 (1.04-1.09) *<0.001*	1.08 (1.04-1.11) *<0.001*	1.07 (1.05-1.09) *<0.001*	1.04 (1.01;1.07) *0.011*

CI, confidence interval; CPAP, continuous positive airway pressure; CRP, C-reactive protein; HR, hazard ratio; MV, mechanical ventilation; PTX3, pentraxin-3.

Using multivariable Cox regression (**Table 2**), baseline PTX3 was an independent predictor of death (per 10 ng/ml, HR 1.08; 95%CI 1.04-1.11; P<0.001) while this did not hold for CRP and D-dimer.

**Figure 2:**
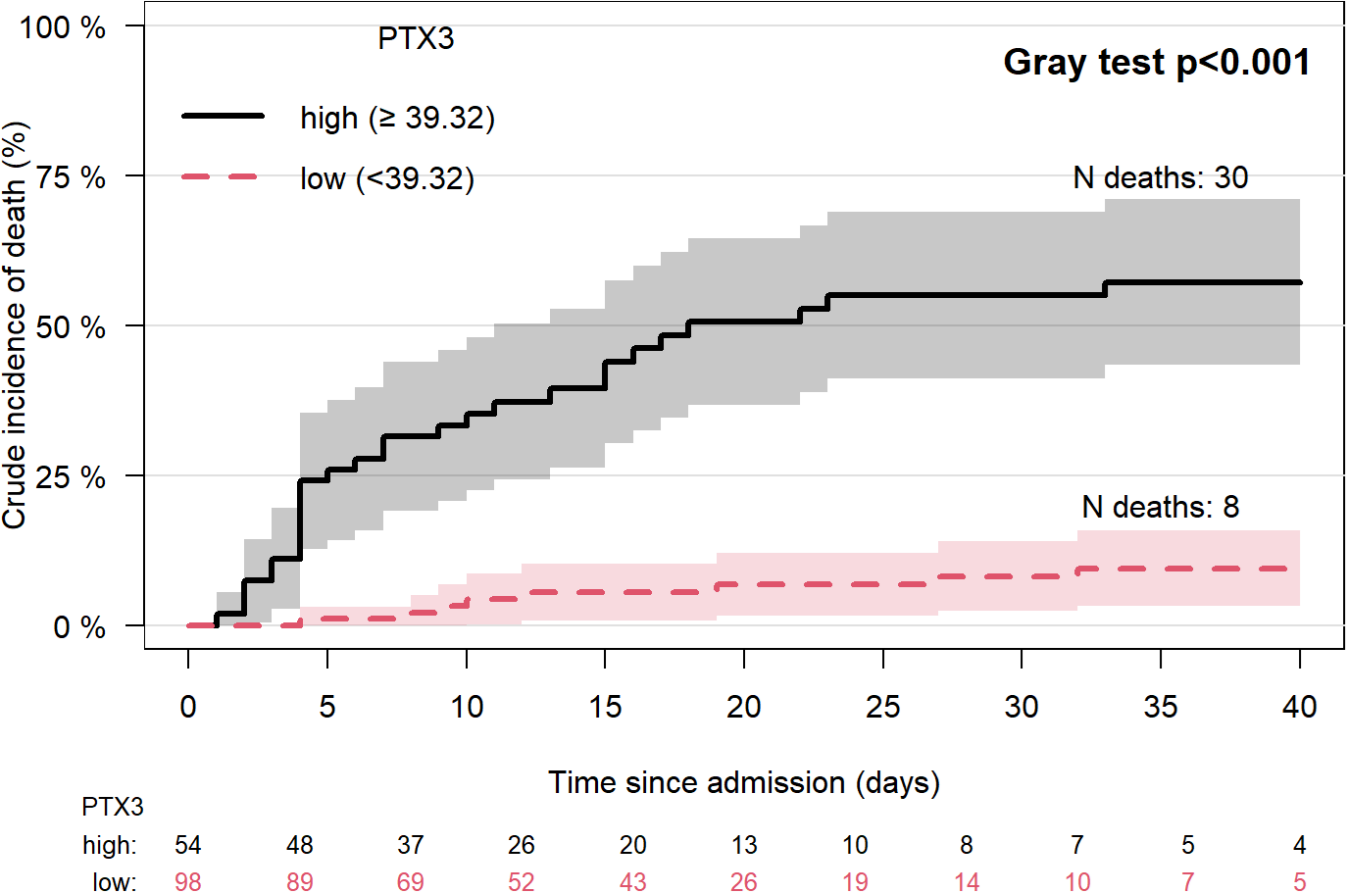
Crude incidence of death (discharge as competing risk) by PTX3 high or low (best cut-off based on Maximally Selected Rank Statistics method). List of abbreviations: N, number; PTX3, pentraxin 3. Lines represent the cumulative crude incidence of the outcome and the shaded areas its 95% confidence interval. The numbers below the plot indicate patients at risk in time in the two groups.

The addition of PTX3 improved the ability of the multivariable Cox-regression model to identify patients that experienced a fatal event during hospitalization as indicated by the C-statistics as a measure of the model discriminatory power. The C-statistic of the model where PTX3 was added to the other covariates increased by 3.7% (from C=74.4 to C=78.1). As compared with CRP levels alone, PTX3 levels alone were better predictors of death within 5 days (time-dependent ROC curves AUC: 86.6 *versus* 74.4), 10 days (78.9 *versus* 68.4) and 30 days (71.1 *versus* 57.3). Patients’ stratification according to PTX3 levels, maximizing the association with mortality (according to Gray test), resulted in the optimal cut-off of 39.32 ng/ml (54 patients [35.5%] had values above the cutoff). **Figure 2** shows crude incidence curves of patients with PTX3 level at admission below or above such threshold. Mortality was significantly higher in the group with higher level of PTX3 (55%, 95%CI: 41-69 vs 8%, 95%CI: 2-14 at 30 days since admission, P<0.001).

**Figure 3:**
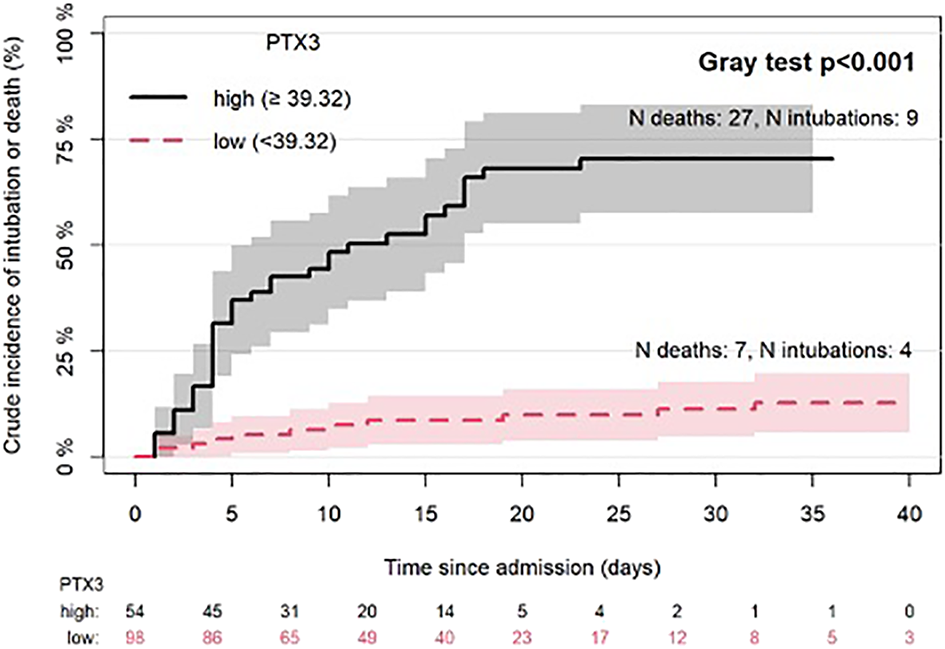
Crude incidence of intubation or death (discharge before intubation as competing risk) by PTX3 high or low (best cut-off based on Maximally Selected Rank Statistics method). List of abbreviations: N, number; PTX3, pentraxin 3. Lines represent the cumulative crude incidence of the outcome and the shaded areas its 95% confidence interval. The numbers below the plot indicate patients at risk in time in the two groups.

Similar results were obtained for the combined endpoint that considers in-hospital death or MV. In this model, PTX3 was associated with the endpoint independently of other considered predictors (HR 1.04; 95%CI 1.01-1.07; P=0.011). Considering dichotomized PTX3 according to the optimal threshold (**Figure 3**), the crude incidence of the combined endpoint was significantly higher in the group with higher level of PTX3 (70%, 95%CI: 58-83 vs 11%, 95%CI: 5-18 at 30 days since admission, P<0.001).()

Moreover, PTX3 plasma level was also associated with risk of thrombotic complication. Fourteen patients with subsequent diagnosis of deep vein thrombosis, arterial thrombosis or pulmonary thromboembolism had significantly higher plasma levels of PTX3 than those without it (median [IQR]: 51.4 [24.6-94.4] *versus* 21 [13.4-55.2]; Mann-Whitney test P=0.049). Although the relatively low number of events did not allow a formal comparison, levels of PTX-3 appeared to be similar across different type of thrombotic events and consistently higher than those measured among patients who had not developed such complication (**Supplementary Figure 2**)

## Discussion

Infection with SARS-CoV-2 causes a wide spectrum of clinical presentations and different disease courses. Certain clinical conditions, such as advanced age, immunesuppression or particular comorbidities, are nowadays well known to be associated with a higher risk of evolution into severe or critical forms. Regardless, the majority of those who get infected, even among those with high risk of progression, have a self resolving infection and never develop the severe form of the disease. Currently, there is no single marker or score able to predict COVID-19 clinical course at presentation neither to identify those at higher risk of critical illness or death after hospital admission. Such marker would be extremely useful to inform risk stratification of patients hospitalized with COVID-19.

In our cohort, mostly composed of patients who were admitted to medical wards and who did not need intensive care at presentation, we found PTX3 measured upon hospital admission to be a strong and reliable marker of clinical progression, with a high power of discriminating patients with risk of negative outcome (death or mechanical ventilation) from survivors, independently of other risk factors. Such finding confirms and expands, on a larger number of patients, previous observations (28–33, 37). Of note, our study was conducted on patients randomly selected from an observational prospective cohort, thus increasing its representativeness and generalizability compared to previous reports.

An optimal and clinically-meaningful cutoff for PTX3, in this context, is yet to be determined. In our cohort, virtually all patients had PTX3 levels largely higher than those of healthy individuals. Nonetheless, a level >39 ng/ml correctly identified two-thirds of those who ultimately needed mechanical ventilation or died within 30 days, with a good specificity (roughly 90%). If confirmed, our findings, together with previous reports (28–33, 37, 38), suggest that PTX3 could be the most reliable single laboratory marker currently available to assess patients with COVID-19. Indeed, its rapid increase in inflammatory conditions, its correlation with the severity of the disease and its short half-life, combined with the good accuracy showed to predict further disease progression, make it a promising marker of COVID-19 severity and prognosis.

Level of PTX3 was moderately correlated with a common marker of inflammation (CRP) or with D-dimer level, a marker of coagulation activity. However, it also resulted to be a predictor of death independently of them. Moreover, the addition of PTX3 to a model already including CRP and D-dimer, significantly improved its ability to identify patients that experienced a fatal event. All these findings suggest that these parameters may reflect different pathways in the pathophysiology of COVID-19 and, if combined, could contribute to refine prognosis in the early stage of the disease.

Plasma and/or respiratory tract PTX3 levels have also been previously associated with higher mortality among patients under mechanical ventilation (29, 38). In addition, a reduction of PTX3 after treatment with immunomodulatory drugs, such as siltuximab, given as treatment for COVID-19, has been associated with improved outcome (39). Whether this is an epiphenomenon of cytokine storm modulation or PTX3 stands in the pathogenic pathway of the lung damage induced by the virus, is unclear. Previous *in vitro* and animal studies suggested that upregulation of PTX3 can, in specific context, sustain inflammation, induce tissue damage or even facilitate viral replication or entry (40–42). Whatever the mechanism, PTX3 merits further evaluation as possible marker of redundant inflammation during COVID-19, to identify patients who could benefit from immunomodulant or immunosuppressive therapy.

Higher PTX3 plasma levels were also associated, in our study, with increased risk of thrombotic events, a frequent complication of COVID-19. This finding is consistent with previous reports of an association between levels of PTX3 and coagulation markers (31). Notably, PTX3 was also found to be highly expressed in vascular endothelium in the lungs of patients died of COVID-19, thus suggesting that PTX3 could represent a marker of endothelial injury induced by SARS-CoV-2 (28). It is still currently debated if all or a subgroup of patients with COVID-19 may benefit from higher than the prophylactic dose of anticoagulation (43). Whether PTX3 could be used, in combination with other coagulation markers, to identify patients who may benefit from such more aggressive approach, merits to be investigated further.

Our study has some limitations that should be acknowledged. First, it lacked of an external validation. Nonetheless, cross-validation was adopted to correct the over-optimism of estimated predictive ability. In addition, since the association between PTX3 and mortality has been reported in previous smaller studies, we believe that our study can be also regarded as an external validation of these findings. Second, the sample size was relatively small, compared to incidence of COVID-19 when the study was conducted. Third, our study was observational and thus we can not exclude unmeasured confounders or other bias that could have influenced our results. For the same reasons, we are unable to assess any causal relationship between PTX3 and clinical events. Similarly, we cannot attribute PTX3 level increases to the severity of SARS-COV-2 infection itself and not to its complications. Finally, our study was conducted when COVID-19 vaccination was not available. Whether our findings extend to vaccinated individuals is unknown, although, in our opinion, there is no biological justification for thinking otherwise.

In conclusion, PTX3 was strongly associated with subsequent risk of death and need for MV among patients hospitalized for COVID-19, across different age groups or stages of the disease and independently of other conditions influencing the prognosis. We hypothesize that PTX3 plasma level, as single markers or included in scores specifically designed for patients COVID-19, may be extremely useful for their prognostic stratification.

## Data availability statement

The raw data supporting the conclusions of this article will be made available by the authors, without undue reservation.

## Ethics statement

The studies involving human participants were reviewed and approved by National Institutional Review Board for COVID-19 Studies (Spallanzani Hospital). The patients/participants provided their written informed consent to participate in this study.

## Author contributions

GL revised and interpreted the data and wrote the first draft of the manuscript. BB and DB contributed to the revised version of the manuscript with important intellectual content. ER, MD’A, LB, and IB contributed to material preparation and data collection. RL, BB, and CG run the laboratory experiment. DB and MV conducted the data analysis and contributed to data interpretation. AB, AM, and PB conceived the study and contributed to its design. All authors critically revised the manuscript and approved its final version.

## Funding

AM was supported by the Italian Ministry of Health for COVID-19 (COVID-2020-12371640). The authors declare that this study received a philanthropic donation from Dolce & Gabbana fashion house. The funder was not involved in the study design, collection, analysis, interpretation of data, the writing of this article, or the decision to submit it for publication.

## Acknowledgments

The COVID-19 STORM Study Group is composed by: *Steering Committee COVID-19 STORM Study*: Giacomo Bellani, Marina Elena Cazzaniga, Giuseppe Citerio, Ernesto Contro, Giuseppe Foti, Fabrizio Luppi; *Phase I Clinical Research Unit*: Nicoletta Cordani, Serena Capici; *Gastroenterology Unit*: Pietro Invernizzi; *Department of Infectious Diseases*: Anna Spolti, Valentina Orsini, Marta Iannace, Alessandro Soria; *Pneumology Unit*: Paola Faverio; *University of Milano-Bicocca*: Silvia Mori, Stefania Galimberti.

## Conflict of interest

AM, BB, and CG receive royalties for reagents related to innate immunity and are inventors of patents related to PTX3 and other innate immunity molecules.

The remaining authors declare that the research was conducted in the absence of any commercial or financial relationships that could be construed as a potential conflict of interest.

## Publisher’s note

All claims expressed in this article are solely those of the authors and do not necessarily represent those of their affiliated organizations, or those of the publisher, the editors and the reviewers. Any product that may be evaluated in this article, or claim that may be made by its manufacturer, is not guaranteed or endorsed by the publisher.

## Supplementary material

Supplementary Figure 1 Correlation matrix between levels of pentraxin 3, C-reactive protein, D-dimer and lymphocyte count. The table shows the correlation between variables. Each cell shows the correlation coefficient (Spearman) between the variables in the headers. P-values of all correlations were <0.01.

Supplementary Figure 2 Baseline PTX3 levels among patients with or without pulmonary embolism **(A)** or deep vein thrombosis **(B)**. P-values are from Mann-Whitney test.
