# Supplementary figures and images for: Long pentraxin 3 (PTX3) levels predict death, intubation and thrombotic events among hospitalized patients with COVID-19
Source: Front Immunol. 2022 Oct 28;13:933960. doi: 10.3389/fimmu.2022.933960 (PMC9651085; doi:10.3389/fimmu.2022.933960)

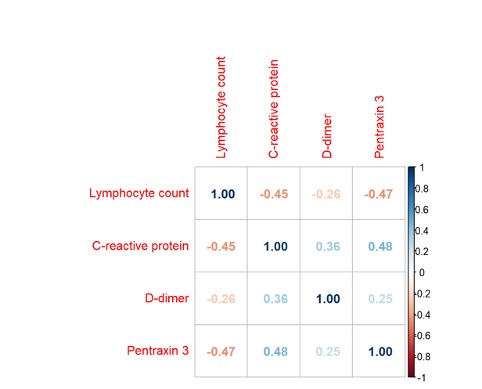

Supplement: Supplementary Figure 1 — Correlation matrix between levels of pentraxin 3, C-reactive protein, D-dimer and lymphocyte count. The table shows the correlation between variables. Each cell shows the correlation coefficient (Spearman) between the variables in the headers. P-values of all correlations were <0.01. [file Image_1.jpeg]

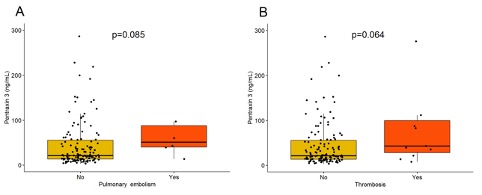

Supplement: Supplementary Figure 2 — Baseline PTX3 levels among patients with or without pulmonary embolism (A) or deep vein thrombosis (B). P-values are from Mann-Whitney test. [file Image_2.jpeg]
